# Natriuretic Hormones, Endogenous Ouabain, and Related Sodium Transport Inhibitors

**DOI:** 10.3389/fendo.2014.00199

**Published:** 2014-12-03

**Authors:** John M. Hamlyn

**Affiliations:** ^1^Department of Physiology, University of Maryland School of Medicine, Baltimore, MD, USA

**Keywords:** salt, sodium, urine, excretion, sodium pump, ouabain, hormone

## Abstract

The work of deWardener and colleagues stimulated longstanding interest in natriuretic hormones (NHs). In addition to the atrial peptides (APs), the circulation contains unidentified physiologically relevant NHs. One NH is controlled by the central nervous system (CNS) and likely secreted by the pituitary. Its circulating activity is modulated by salt intake and the prevailing sodium concentration of the blood and intracerebroventricular fluid, and contributes to postprandial and dehydration natriuresis. The other NH, mobilized by atrial stretch, promotes natriuresis by increasing the production of intrarenal dopamine and/or nitric oxide (NO). Both NHs have short (<35 min) circulating half lives, depress renotubular sodium transport, and neither requires the renal nerves. The search for NHs led to endogenous cardiotonic steroids (CTS) including ouabain-, digoxin-, and bufadienolide-like materials. These CTS, given acutely in high nanomole to micromole amounts into the general or renal circulations, inhibit sodium pumps and are natriuretic. Among these CTS, only bufalin is cleared sufficiently rapidly to qualify for an NH-like role. Ouabain-like CTS are cleared slowly, and when given chronically in low daily nanomole amounts, promote sodium retention, augment arterial myogenic tone, reduce renal blood flow and glomerular filtration, suppress NO in the renal vasa recta, and increase sympathetic nerve activity and blood pressure. Moreover, lowering total body sodium raises circulating endogenous ouabain. Thus, ouabain-like CTS have physiological actions that, like aldosterone, support renal sodium retention and blood pressure. In conclusion, the mammalian circulation contains two non-AP NHs. Identification of the CNS NH should be a priority.

## Introduction

Natriuretic hormones (NHs) can be defined as substances whose circulating levels and effects fluctuate in a parallel manner with dietary sodium intake (1). NHs have long been implicated in sodium balance and are likely to be of the most significance in western acculturated societies where sodium intake typically is >100 meq/day (2). Indeed, ingestion of high salt meals raises the osmolarity of the circulation, stimulates secretion of antidiuretic hormone (ADH), and raises the natriuretic activity of the blood. In principle, the mode of action of NHs includes suppression of primary active sodium transport in the kidney and/or damping of secondary active transport systems involving sodium (1) or even potassium (3), effects on renal vascular tone and glomerular filtration rate (GFR), and activation of intrarenal natriuretic factors, such as prostaglandins, nitric oxide (NO), or dopamine. This article presents a personal and condensed overview of known and unknown non-atrial NHs and addresses the role of endogenous sodium pump inhibitors as NHs.

## Searching for Natriuretic Hormones

It is well accepted that sodium balance is not fully explained by the up and downregulation of glomerular filtration and mineralocorticoid-stimulated reabsorption (4, 5). The first clear evidence for a “third factor” arose from the pioneering experiments of deWardener in which dogs that received excess mineralocorticoid and vasopressin increased their urinary sodium excretion in response to blood volume expansion with saline at a time when glomerular filtration was being lowered experimentally (6). Thus, the increase in sodium excretion was mediated by diminished tubular reabsorption of sodium and water. Cross-circulation studies, as well as work using isolated kidney studies in dogs and rats (6–10) excluded significant alterations in the composition of the blood, changes in renal nerve activity, glomerular filtration, renal blood flow, or renal perfusion pressure as mediators. A humoral “NH” was required.

The discovery of the atrial peptides (APs) and their natriuretic activity initially promised to explain some of the outstanding functions of an NH (11–13). APs augment sodium excretion (14–16) and saline infusions raise plasma AP (17, 18). However, in dogs, the effects of physiological changes in plasma APs and low dose infusions on sodium excretion were less obvious and, under certain experimental conditions, circulating APs and sodium excretion changed diametrically or, were temporally unconnected (19–21). Thus, some other NH was required.

The search for humoral agents that trigger salt excretion has relied on a variety of assays that range from isolated enzymes all the way to whole kidneys and animals (22). Table 1 lists some tissues and fluids from which a variety of natriuretic factors were obtained. It is a significant accomplishment that numerous factors with natriuretic activity including guanylin, uroguanylin, urodilatin, LLu-α, xanthurenic acid, and a number of steroidal sodium pump inhibitors have been isolated and identified (14, 22–27). These materials likely account for some of the bioactivity in some, but not all, studies where natriuretic activity has been demonstrated. It is less clear that any of these materials fits the physiological profile expected for a NH as will be apparent from the discussion that follows.

**Table 1:** **An overview of sources and characteristics of natriuretic factors**.

Source for isolation	Characteristics	References
Adrenal	No short acting factors described	(8, 28, 29)
	Ouabain,^a^ proscillaridin A-like compound^b^	(30)
Blood	Rapid onset, chymotrypsin-sensitive	(31–34)
	Rapid sustained natriuresis, MW < 500–700	(35)
	Trpysin sensitive, slow onset	(36)
	Precursor? slow onset	(37)
	Leucine aminopeptidase-sensitive, chymotrypsin-resistant	(38–41)
	Ouabain^a^	(42)
Hypothalamus/pituitary	ADH, Oxytocin, MSH	See text
	Ouabain^a^	(43)
Intestine	Guanylin (small heat stable peptide)	(26, 44)
Kidney	High MW, release PGE_2_ dependent	(45–48)
	Urodilatin^c^ (ANP 95–126) Small peptide	(24, 49, 50)
Liver	Long acting, high MW (bound?), hepatic blood > portal blood	(51–59)
Urine	Low MW, Chymotrypsin-sensitive peptide	(33)
	Low MW, non-peptidic, acidic, Sephadex post salt fraction	(60–62)
	LLU-α^d^	(3)
	High MW, slow onset	(36, 46, 63–65)
	Marinobufagenin^e^	(66)
	Prolidase-sensitive peptide	(61)
	Urodilatin^c^ (small peptide)	See kidney
	Uroguanylin (small heat stable peptide)	(27, 44)
	Xanthurenic acid β-glucoside and xanthurenic acid sulfate	(25, 67)

*MW, molecular weight*.

*All materials listed with high MW are likely proteins*.

*^a^Natriuretic at supraphysiological and pharmacological doses*.

*^b^Expected to have similar natriuretic activity as the bufadienolides (68, 69)*.

*^c^Not likely to circulate in significant amounts*.

*^d^LLU-alpha; 2,7,8-trimethyl-2-(pcarboxyethyl)-6-hydroxychroman*.

*^e^Immunoreactivity present in the circulation (70) but not isolated from blood. The natriuretic effect of MBG *per se* has not been reported but is inferred from studies with bufalin and closely related steroids (68)*.

## Natriuretic Hormones: How Many?

Other than the APs, there are numerous hormones and endogenous materials that are known natriuretic agents. These include melanocyte stimulating hormone, dopamine, certain phospholipids, prostaglandins, kinins, and parathyroid hormone (71). These are not discussed here.

Evidence based upon pharmacological interventions, as well as an analysis of the kinetics of salt excretion mentioned below, suggests there are at least two major NH mechanisms unrelated to the APs. One mechanism is activated by the central nervous system (CNS) and the other involves maneuvers that increase atrial stretch. Pharmacological inhibition of renal NO blunts the magnitude of saline natriuresis (72) and both specific and non-selective dopamine antagonists attenuate volume expansion and water immersion (i.e., atrial stretch mediated) natriuresis but not that activated by CNS sodium (73–77). Yet another key factor that distinguishes these two NH systems is their kinetics; the rates of the decline in sodium excretion when the natriuretic stimuli are abruptly removed differ markedly for CNS- and atrial distention natriuresis. The kinetic features are potentially diagnostic; they can be used to evaluate candidate NHs.

**Figure 1:**
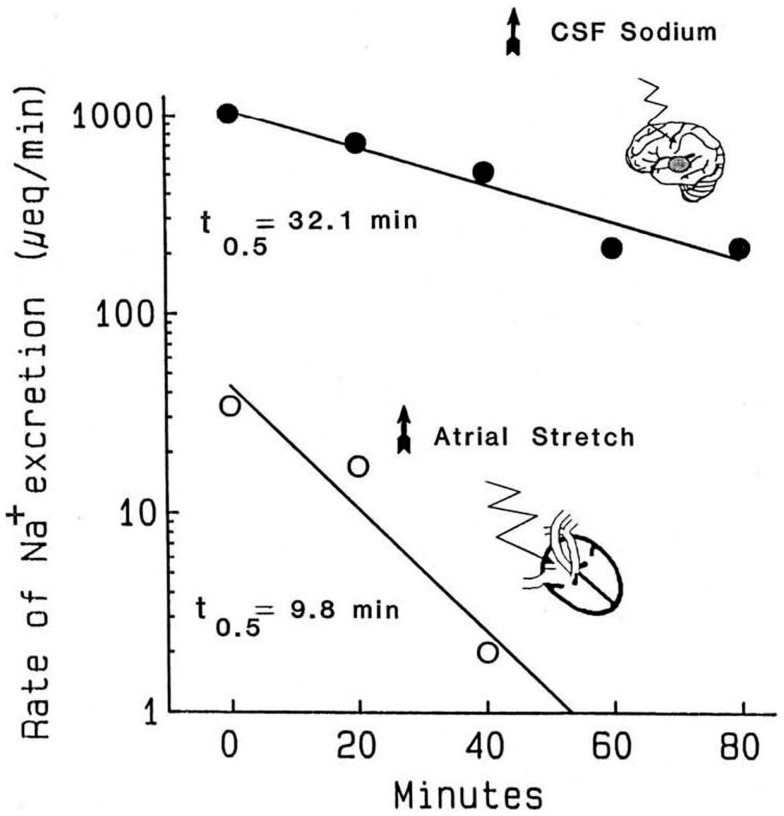
**Kinetics of the decline in natriuresis following abrupt removal of the natriuretic stimulus**. Reproduced from Ref. (71) with permission. Data are adapted from Ref. (21) in conscious dogs and Ref. (86) in conscious goats. Reported values for the *t*_1/2_ of the decline in CNS natriuresis range from 24 to 32 min in the goat, 32 min in the sheep, and 15 min in the rat (71). These half times are likely to represent the clearance of their respective humoral mediators from the circulation.

The atrial distention arising from balloon inflation requires intact cardiac but not renal nerves, the stretch can be reversed in seconds, and the evoked natriuresis declines rapidly (21). Critically, the kinetics of the decline in natriuresis are uncontaminated by residual volume that typically would remain following a saline load (78). The second experimental paradigm is the natriuresis evoked by infusion of hypertonic saline into the brain. As the flow rates in the cerebral ventricles are much higher than the rates at which hypertonic stimuli are typically infused, simply stopping the infusion exposes the kinetics of the decline in salt excretion. Accordingly, Figure 1 compares the decline in renal sodium excretion evoked by either atrial distension or CNS sodium. Three points are apparent: (1) the decay kinetics in both instances are first order; for CNS natriuresis, they remain linear for well over 1 h. The kinetics demonstrate that a single reaction likely is the dominant rate limiting step for the natriuresis evoked by each stimulus. (2) The CNS natriuresis, when activated by hypertonic saline (79–83), dehydration (84), or norepinephrine (85), produces similar rate constants with no major species differences. (3) The rate constants for the decline in CNS natriuresis are ~2–3-fold less (slower) than that evoked by atrial distension. Thus, the combined evidence derived from the sensitivity to pharmacological agents and the kinetic observations indicate that CNS- and atrial distension natriuresis must be mediated by different mechanisms.

Compensatory mechanisms might conceivably alter the kinetics in Figure 1, especially if significant salt and water loss were to occur along with declining blood pressures. During the 40 min atrial distention in Figure 1, blood pressure increased modestly. Plasma renin was suppressed in one set of experiments but not another. Following the distension, in one set of experiments, blood pressure remained elevated even though the natriuresis declined rapidly and aldosterone was unchanged or increased. Nevertheless, changes in aldosterone would have been too slow to have had impact. Under the conditions used, and among the measured hormonal and hemodynamic variables, the only changes convincingly associated with the decline in natriuresis following atrial distension were the return of left, right, and pulmonary pressures (i.e., cardiac nerve activity) to normal. With regard to CNS natriuresis, the decline in natriuresis is an extended first-order process; the absence of curvature over the time course implies no major influence by a compensatory process.

Among the candidate NHs in Table 1, there is, unfortunately, no readily interpretable information regarding the halftimes for the decline in their natriuretic effects. Most of the unidentified materials were impure, with variable onset times, and, reminiscent of urodilatin (49), some produced a natriuresis that lasted many hours following infusion. The absence of kinetic information is understandable; the primary experimental emphasis was the demonstration of natriuresis *per se*. And for decay kinetics to be informative, a near steady-state natriuresis would ideally be desirable prior to stimulus removal. This is not always an easy condition to meet. Regarding the recently identified materials in Table 1, no kinetic information is available. However, among all the materials, urodilatin shows a most interesting physiological correlate; in human beings, urinary urodilatin excretion closely paralleled the circadian rhythm for sodium excretion over many days (49). As urodilatin itself is not found in the circulation, it is not, by definition, an NH; although the unknown substance (?) that presumably links sodium intake with urinary urodilatin and sodium excretion could be. Thus, for all listed materials in Table 1, there is currently no compelling evidence that their behaviors fit the definition of a physiologically relevant NH given in the introduction.

Hereafter, I focus primarily on CNS natriuresis and consider the potential role of sodium pump inhibitors as NHs.

## CNS Natriuresis

The brain, via an unknown humoral NH, mediates the natriuresis evoked by increased plasma sodium concentration, intracerebroventricular (icv) sodium, and dehydration (79, 81, 87). The natriuresis may be damped but is not eliminated by renal denervation (88), is activated by small increases of plasma sodium (1–2 mM). The CNS NH may have a dominant influence in postprandial natriuresis (89) and is a blood-bourne factor distinct from APs (90, 91), ADH (92), or dopamine (74).

Central nervous system natriuresis can be activated by the elevation of either blood-bourne or cerebrospinal fluid sodium; both dehydration and postprandial natriuresis are blocked or reversed by hyponatremic CSF (93–95) or rehydration (96). Push–pull perfusion techniques suggest a discrete area of the third ventricle is near the sodium sensing apparatus (97). Further, the ablation of central structures, including the anteroventral and posterior hypothalamus in a variety of species, or decapitation, profoundly influence the ability to regulate osmotic balance, tolerate hyperosmotic challenge, and excrete sodium (80, 83, 98–106). The lesioned areas have included the median eminence, medial preoptic nucleus, organum vasculosum of the lamina terminalis, and the periventricular preoptic area. The consequences of these lesions are impaired thirst and ADH secretion, reduced renal natriuretic response, and hypernatremia. In contrast, this same system, when overactivated, can lead to profound hyponatremia. This phenomenon, sometimes termed “cerebral salt wasting,” and resembling some of the features of the syndrome of inappropriate ADH secretion, has been noted in some CNS disorders (107–111).

The observation that dehydration results in hypernatremia and provokes a compensatory natriuresis in the face of reduced extracellular fluid volumes, and that the natriuresis subsides with rehydration, suggests that the tendency to hypernatremia during dehydration and following a high salt meal is actively opposed by an unknown osmotically sensitive mechanism [see in Ref. (84)]. In each instance, the natriuretic response to these stimuli is present in animals with denervated kidneys but absent in animals with hypothalamic lesions (84, 112). Further, CNS natriuresis is not explained by blood pressure changes and persists when renal artery pressures are servo controlled (92, 113).

In each of the aforementioned situations, changes in circulating ADH have been implicated as the efferent mediator of CNS natriuresis (114, 115). Indeed, CNS natriuresis is either absent or slowed in rats congenitally deficient in AVP (116, 117), is absent in hypophysectomized rats but reappears in rats pretreated with large amounts of ADH and in rats given a dD-AVP analog (88). ADH certainly contributes to the control of sodium excretion in rats, dogs, and man (84, 89, 118–123). ADH infusions are natriuretic, and specifically implicated in CNS (114, 115) but not saline natriuresis. However, ADH is not sufficient to account for CNS natriuresis (92, 117), although it may be permissive (124, 125). For example, AV3V-lesioned sheep and dehydrated normal sheep both lost similar amounts of body water, although the hypernatremia was much worse in the lesioned animals (112). Thus, something other than ADH was lacking in the lesioned animals to explain the greater hypernatremia with the same overall water loss.

Little is known about the chemical nature of the CNS NH other than it appears to be heat stable (126). Its actions have an interesting temporal association with ADH and/or oxytocin (92, 127). For example, plasma ADH rises during the prehypertensive period associated with mineralocorticoid escape; a period when increased CNS NH would be expected (128). Consistent with the latter supposition, urinary sodium excretion in sheep given 3–4 day infusions of aldosterone was almost entirely blocked by the acute CNS administration of a low sodium cerebrospinal fluid during mineralocorticoid escape (129). Further, mineralocorticoids also augment the osmotic sensitivity of ADH secretion (130).

Oxytocin also has a role in renal sodium excretion (131–134) and restores the ability of hypophysectomized dogs and rats to excrete sodium at a brisk rate during saline expansion (20, 135). Yet other humoral factors implicated during CNS and ADH natriuresis include an inhibitor of prostacyclin synthesis (136) and a humoral substance that inhibits active sodium transport in toad bladder (137). Hemorrhage, paradoxically, also evokes a natriuresis that depends on the CNS (118). The natriuresis is blocked when intrarenal prostaglandin synthesis is inhibited (119). The simplest interpretation is that activation of intrarenal V1 receptors stimulates prostaglandin synthesis and the resultant products influence sodium reabsorption at distal tubular sites (138). Overall, the phenomenon ascribed to CNS natriuresis has complex interdependencies and is associated with the diminution of renal tubular sodium transport.

Of significant relevance, CNS-mediated natriuresis depends upon the prevailing level of dietary sodium intake. In sodium depleted dogs, infusion of hypertonic saline into the carotid artery is not natriuretic (139). Moreover, the phenomenon of postprandial natriuresis in the sheep is activated only when dietary sodium intakes reach a threshold of 50–75 mmol of sodium/24 h (140), i.e., when plasma renin and aldosterone are largely suppressed. Thus, the CNS NH system is likely of great physiological relevance; it is appropriately integrated with other key factors that govern long-term sodium balance.

The CNS also has a permissive role in the response to saline expansion of blood volume (79, 102, 103). Hypophysectomy reduces saline natriuresis; the deficit is reversed partially by administration of oxytocin and ADH (141). Furthermore, the application of a constricting vice to the neck of anesthetized dogs so as to exclude the brain and pituitary factors from the circulation impairs saline natriuresis (102). In view of the abovementioned role of the CNS, it is surprising that remarkably little attention has been focused on the natriuretic activity associated with extracts from brain and pituitary (Table 1). The little that is known is that the bioactivity of natriuretic extracts from hypothalamus persists following treatment with thioglycollate (to exclude oxytocin or ADH), and that an unidentified tridecapeptide was found in bioactive fractions from the posterior pituitary (142, 143).

## Are Sodium Pump Inhibitors Natriuretic Hormones?

There is much evidence linking sodium pump inhibitors with salt balance and cardiovascular and renal disease (144, 145). The Na,K-ATPase inhibitory activity of plasma from normal individuals on a high sodium diet was 25 times greater than that when the individuals were on a low sodium intake (146). Further, the plasma from individuals on high sodium diets, purified natriuretic material from urine, and ouabain, all stimulated glucose-6-phosphate dehydrogenase (G6PD) activity. G6PD activity is claimed to be inversely related to Na,K-ATPase activity (147) and related to inhibition of proximal tubular Na,K-ATPase (148), although the G6PD assay is not considered a surrogate method for the Na,K-ATPase.

Nevertheless, increased blood levels of sodium pump inhibitors, as measured by traditional well-accepted means, have been repeatedly associated with acute volume expansion, high dietary salt, mineralocorticoid excess, chronic renal failure, and CNS natriuresis (31, 32, 35, 38, 149–155). Haddy and coworkers using animal models of low renin hypertension observed that sodium pump inhibition could be reproduced in normal animals given a rapid volume expansion and that this effect could be transferred to the arteries of another animal via the plasma (156). Further, in acutely saline-expanded dogs, the plasma levels of a polar Na,K-ATPase inhibitor and a digoxin immunoreactive material were elevated at a time when endogenous ouabain (EO) was unchanged (37, 157). Moreover, the plasma of dogs undergoing atrial distension strongly inhibited the ouabain-sensitive ^86^Rb uptake into human red cells. Notably, the bioactivity of the plasma declined substantially when retested a few days later, and was undetectable after 10 days (Hamlyn and Goetz, unpublished observations). This indicates that the inhibitor is unstable in plasma and is reminiscent of the labile digoxin-like material described by Graves et al. (158). Other work implicated the CNS in the control of humoral sodium pump inhibitors; Buckalew et al. (159) found that the jugular effluent inhibited active sodium transport to a greater extent than the blood from the femoral vein. Further, increased levels of circulating sodium pump inhibitors depend upon the integrity of hypothalamic structures within the AV3V area (103, 160). Moreover, the lesion sites overlap those whose integrity is required for CNS natriuresis. Thus, the interrelationship between increased circulating sodium pump inhibitors and natriuresis continues to be of interest. When taken together, there is no doubt that the circulation contains inhibitors of sodium transport, but what are these materials, do their levels change appropriately with salt, and are they natriuretic? Below we focus on sodium pump inhibitors that have been isolated and that have been previously linked with the aforementioned criteria.

## Identification of Sodium Pump Inhibitors

Starting from either human plasma or urine (brain, adrenal, and the eye are not discussed here), four groups isolated sodium pump inhibitors and identified them as ouabain- (42, 161, 162), digoxin- (163), marinobufagenin- [MBG, (66)], and telocinobufagin-like steroids (162, 164), respectively. There are altered levels of these materials in numerous experimental and clinical studies (70, 164–169). All these steroids inhibit the sodium pump and, when bound, at least one evokes biased signaling in a manner strikingly reminiscent of the β-adrenergic receptor (168, 170–172). These cardiotonic steroids (CTS) typically are natriuretic and variably kaliuretic when infused acutely at pharmacological (micromolar) doses into anesthetized animals or the renal artery and, in the case of ouabain, selectively inhibit sodium transport in the distal tubules (68, 69, 173, 174). The natriuretic response is linearly related to the inhibition of Na pumps in the dog (175). But are they physiologically relevant NHs?

## What Do the Kinetics of the Decline in Natriuresis Tell Us about the Role of Known Sodium Pump Inhibitors?

By comparing the circulating half lives of any putative NH with the half times in Figure 1, it is possible to determine whether it is a plausible mediator of natriuresis. Here, I examine the circulatory half lives of a number of well-known sodium pump inhibitors and compare them with the information in Figure 1. For example, in the dog, the plasma half lives for intravenous ouabain, digoxin, resibufagenin, and bufalin were ~18 h, ~30 h, 21 min, and 25 min, respectively [Ref. (176–178)]. In the rat, the circulating half lives for intravenous cinobufagin, resibufagenin, and bufalin were 44, 42, and 25 min, respectively (179). Therefore, it is apparent that, among these known steroidal sodium pump inhibitors all, with the exception of bufalin, are simply cleared too slowly from the circulation to be kinetically plausible humoral mediators of CNS natriuresis. In the case of atrial distention natriuresis, the kinetic analysis reveals that none of the abovementioned sodium pump inhibitors are likely primary humoral mediators. With regard to CNS natriuresis, only the clearance of bufalin is sufficiently fast in both dogs and rats to warrant further investigation. The kinetic analysis does not prove bufalin as the humoral mediator in CNS natriuresis, but simply suggests that this steroid (or those that are closely related but for which no clearance data are available, e.g., MBG) cannot, as yet, be excluded. A lingering concern with bufalin, or any CTS sodium pump inhibitor, as a NH is the potentially serious conceptual problem that their acute vasoconstrictive action within the renal vasculature will oppose their tubular effects (180).

## Renal Sodium Pump Isoforms: Is Their Ouabain Sensitivity Important?

Nearly all mammalian tissues express the α-1 catalytic subunit of the sodium pump; muscle and muscle and nerve also express sodium pump isoforms with α-2 and α-3 subunits (181). In the rat kidney, sodium pumps with the α-1 catalytic subunit are insensitive to micromolar ouabain but are somewhat sensitive to bufalin and marinobufagenin; the acute natriuretic effect of bufalin is greater than that of ouabain (69). For many years, it was believed that the kidney expressed only the α1 isoform even though the ouabain sensitivity of the renal Na pump increases progressively along the nephron (182); the distal tubules are believed to be ~50–100-fold more sensitive than their proximal tubule counterparts. More recently, small numbers of highly ouabain-sensitive α-2 sodium pumps have been detected in rat kidney and they are functionally significant. For example, in response to acute low doses of ouabain, the α2 sodium pumps trigger enhanced Ca^2+^ signaling and NO generation in the descending vasa recta (183). It is not known if these signaling effects extend to the renal epithelia, but if they do then the acute natriuretic effects of ouabain could involve short-term NO-mediated events. In contrast, the acute natriuretic effects of bufalin and other bufadienolides are thought to be mediated by inhibition of α1 sodium pumps (184).

In the kidney, the renal ouabain-insensitive α-1 sodium pumps far outnumber their ouabain-sensitive α-2 cousins. Interestingly, saline natriuresis was augmented when rodent α-1 sodium pumps were made highly ouabain-sensitive (185). Further, the augmented component of the natriuresis was blocked by digoxin antibody fragments (Fab). However, the kinetic analysis in Figure 1 makes it clear that neither ouabain nor digoxin are viable mediators of atrial distention (saline) natriuresis; the digoxin Fab fragments must, therefore, have interacted with an unknown material that preferred ouabain-sensitive sodium pumps. Thus, occupation of the ouabain binding site by this material can contribute to, but does not fully account for, the phenomenon of saline natriuresis.

## Ouabain as a Salt Retaining Steroid

In contrast to the well-accepted acute natriuretic effects of high doses of sodium pump inhibitors, the chronic effects of low concentrations can be diametrically opposite. In the case of ouabain, the prolonged daily administration of low nanomole amounts in the rat suppresses Ca^2+^ signaling and NO generation in the endothelium of the descending vasa recta, reduces renal blood flow and glomerular filtration, raises sympathetic nerve activity, directly augments vascular myogenic tone and contractility, and raises blood pressure (186–193). Further, chronically reduced total body sodium in human beings is associated with elevated circulating levels of EO (194, 195), i.e., the chronic relationship between plasma EO and salt intake is, like aldosterone and renin, roughly “L”-shaped (196). In addition, and as might be anticipated from the above noted chronic observations, clinical studies have shown that among salt-loaded EH patients, renal tubular sodium reabsorption was highest in the group with elevated circulating EO (197). Thus, the behavior of circulating EO under physiological circumstances, as well as its long-term vascular and renal tubular actions, all appear to favor sodium retention.

Dramatic increases in circulating EO have been reported during exercise, a state associated with increased sympathetic activity and a decline in renal blood flow (198). The circulating levels of EO rise acutely in response to the stress of cardiac surgery (199) and the preoperative plasma levels of EO enhance the identification of those patients who will develop acute kidney injury postsurgery (200). Once again, the behavior and actions of EO in these stressful situations is associated directly or indirectly with salt and water retention, rather than salt excretion. When taken together, the current evidence strongly favors the view that EO is a physiologically relevant hormone with a variety of interesting actions that augment vascular tone and promote renal sodium retention.

In summary, the hunt for NHs has led recently to the complete identification of numerous natriuretic materials. In spite of these notable successes, none of the materials seems to fit the anticipated physiological profile for a mammalian NH. Much evidence indicates there are two major non-AP NHs that remain to be isolated and identified. It may be argued that identification of the CNS NH should be a priority in view of its broad physiological relevance, relationship to dietary sodium intake, and the implication of a profound role in salt balance in a number of pathological disorders.

## Conflict of Interest Statement

The Review Editor Frans H. H. Leenen declares that, despite having collaborated with the author John Hamlyn, the review process was handled objectively and no conflict of interest exists. The author declares that the research was conducted in the absence of any commercial or financial relationships that could be construed as a potential conflict of interest.
